# Therapeutic effects of natural compounds against diabetic complications via targeted modulation of ferroptosis

**DOI:** 10.3389/fphar.2024.1425955

**Published:** 2024-09-18

**Authors:** Zhen Zhang, Luxin Li, Wei Fu, Zhengchao Fu, Mahang Si, Siyu Wu, Yueying Shou, Xinyu Pei, Xiaoyi Yan, Chenguang Zhang, Tong Wang, Fei Liu

**Affiliations:** ^1^ Heilongjiang Key Laboratory of Anti-Fibrosis Biotherapy, Mudanjiang Medical University, Mudanjiang, China; ^2^ School of First Clinical Medical College, Mudanjiang Medical University, Mudanjiang, China; ^3^ Public Health School, Mudanjiang Medical University, Mudanjiang, China

**Keywords:** ferroptosis, natural products, diabetic complications, regulatory cell death, pathogenesis

## Abstract

Diabetes mellitus, a chronic metabolic disorder, can result in serious tissue and organ damage due to long-term metabolic dysfunction, leading to various complications. Therefore, exploring the pathogenesis of diabetic complications and developing effective prevention and treatment drugs is crucial. The role of ferroptosis in diabetic complications has emerged as a significant area of research in recent years. Ferroptosis, a recently discovered form of regulated cell death closely linked to iron metabolism imbalance and lipid peroxidation, has garnered increasing attention in studies exploring the potential role of natural products in its regulation. This review provides an overview of the mechanisms underlying ferroptosis, outlines detection methods, and synthesizes information from natural product databases. It also summarizes current research on how natural products may regulate ferroptosis in diabetic complications. Studies have shown that these products can modulate the ferroptosis process by influencing iron ion balance and combating oxidative stress. This highlights the potential of natural products in treating diabetic complications by regulating ferroptosis, offering a new strategy for managing such complications.

## 1 Introduction

According to a report by the International Diabetes Federation, there will be approximately 536.6 million diabetic patients worldwide in 2021, with the number expected to rise to 783.2 million by 2045 (Sun et al., 2022). Diabetes is characterized by hyperglycemia, which triggers various metabolic signaling pathways, causing inflammation and affecting cytokine secretion. Hyperglycemia is also a major contributor to the development of various diabetic complications (Volpe et al., 2018; Zhou et al., 2019), which are the most disabling and fatal consequences for diabetic patients. Hyperglycemia arises from multiple organ dysfunction. Hyperglycemia can lead to renal tubular metabolic disorders, oxygen deficiency, oxidative stress, and programmed cell death, ultimately promoting renal tubular damage and renal fibrosis. This cascade of events results in diabetic kidney disease (DKD) (Shu et al., 2023; Yang et al., 2023a). Hyperglycemia-induced oxidative stress can also lead to diabetic retinopathy (DR) through mitochondrial defects, apoptosis, inflammation, lipid peroxidation, and changes in structure and function (including abnormal microcirculation and neural degeneration). Furthermore, hyperglycemia, dyslipidemia, and altered insulin signaling cause a variety of pathological changes in neurons, glial cells, and vascular cells, leading to neurological dysfunction and, ultimately, neuropathy. In summary, diabetic complications inflict multifaceted harm, not only increasing the complexity of the disease and the difficulty of treatment but also significantly raising the risk of disability and death in patients. Despite recent advancements in the treatment of diabetes and its complications, their incidence continues to rise. Therefore, the discovery of novel treatment approaches remains crucial.

Ferroptosis, a caspase-independent form of regulated cell death, is specifically triggered by intracellular lipid peroxidation and tightly regulated by glutathione peroxidase 4 (GPX4) (Li et al., 2020). It disrupts iron, amino acid, and lipid metabolic processes, leading to the accumulation of reactive oxygen species (ROS) and ultimately impacting disease progression (Liu et al., 2020; Jiang et al., 2021). Notably, a close link exists between diabetic complications and ferroptosis. Hyperglycemia can induce iron overload, and this iron imbalance promotes ROS production and oxidative stress, ultimately culminating in ferroptosis (Musheshe et al., 2022). Compared to traditional cell death pathways, ferroptosis exhibits a strong association with iron accumulation and oxidative stress (Li L. et al., 2023). Therefore, targeting ferroptosis regulation holds promise for the development of novel therapeutic strategies for diabetes and its complications.

Naturally occurring compounds derived from plants, microbes, or animals have served as a cornerstone in the treatment of various diseases for centuries. Research has demonstrated that certain natural compounds possess cytoprotective properties, including the ability to mitigate oxidative stress and improve the course of associated pathologies (Baek et al., 2015; Vazquez Olivo et al., 2019). The role of oxidative stress in the development of diabetic complications is well-established, leading to a surge in research on the potential therapeutic application of natural products in managing diabetes and its complications. In this context, excessive activation of ferroptosis has been implicated in the pathological processes of diabetic complications, including cell damage and the inflammatory response (Yu et al., 2021). In light of this, the application of natural products for regulating ferroptosis has become a research direction of great interest. Certain natural products are believed by researchers to play a positive role in regulating iron metabolism and cell death. These products may act as natural ferroptosis inhibitors by reducing ROS production, regulating iron homeostasis, binding ferrous iron, and inhibiting the degradation of GPX4. Additionally, they may exert their pharmacological effects through mechanisms that regulate ferroptosis-related signaling receptors and pathways (Zhang S. et al., 2021). Therefore, natural products that modulate iron metabolism and ferroptosis offer a potential therapeutic strategy for patients with diabetes and diabetic complications.

The research on the mechanism of ferroptosis and the exploration of the application of natural products provide new perspectives and possibilities. However, the complex components and diverse sources of natural products make it difficult to predict drug interactions, which limits the clinical effects of natural products. Therefore, in the development of natural products as ferroptosis modulators, improving drug efficacy as well as determining the most applicable treatment regimen will be the focus of future research. This review provides an in-depth examination of the discovery process of ferroptosis and its regulatory mechanisms, aiming to enhance our understanding of this form of cell death. In addition, we compiled a natural product database and summarized the effects of these products on ferroptosis regulation and their potential impact on diabetic complications. Bioactive components within certain natural products have been shown to modulate ferroptosis through diverse mechanisms. By exploring this burgeoning field of research, we hope to establish a novel theoretical foundation for the development of more efficacious and safer treatments, ultimately providing new insights into the management of diabetic complications.

## 2 Overview of ferroptosis

### 2.1 The discovery of ferroptosis

**FIGURE 1:**
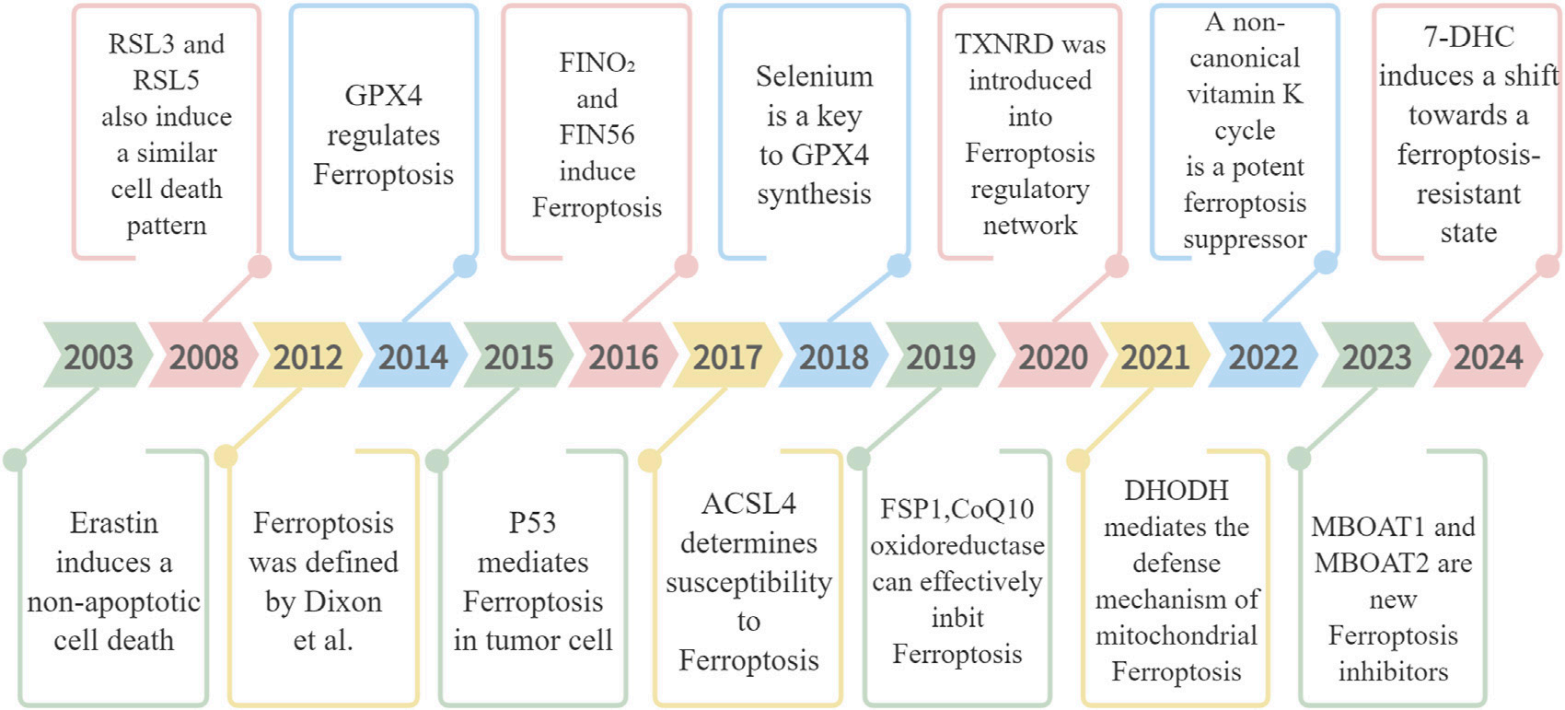
The discovery process of ferroptosis, focusing on the molecules and regulators involved.

Ferroptosis has emerged as a major area of research focus in recent years, with ongoing investigations into its intricate molecular mechanisms (Figure 1).

In 2003, Sonam Dolma *et al.*, working in the laboratory of Brent R. Stockwell, discovered a new compound, Erastin, capable of selectively inducing cell death in tumor cells harboring mutated *RAS* genes. The cell death it induced exhibited a novel, non-apoptotic character (Dolma et al., 2003). Building upon this research, Wan Seok Yang and Brent R. Stockwell further identified two new compounds, RSL3 and RSL5. Similar to Erastin, these compounds could induce iron-dependent, non-apoptotic cell death in tumor cells with mutant RAS. Notably, this cell death could be inhibited by deferoxamine mesylate (DFOM) and antioxidants (vitamin E) (Yang and Stockwell, 2008). It was not until 2012 that this form of cell death was officially named ferroptosis (Dixon et al., 2012).

A pivotal discovery in 2014 by Yang *et al.* established GPX4 as a critical ferroptosis regulator (Yang et al., 2014). GPX4’s role lies in metabolizing lipid oxides through a glutathione (GSH)-dependent reaction, thereby preventing ferroptosis by inhibiting lipid peroxide accumulation. Research has also identified other important regulatory factors. For instance, siderophore proteins like transferrin and glutamine are essential for ferroptosis induction. Additionally, lipoxygenase (LOX) catalyzes the oxidation of polyunsaturated fatty acids (PUFAs) through a phosphorylase kinase G2 (PHKG2)-dependent iron pool, promoting ferroptosis. Conversely, compounds with reductase activity (RTA) impede ferroptosis by blocking lipid autooxidation (Jiang et al., 2015). The year 2015 marked another significant development when Gu Wei’s team revealed the tumor-suppressive role of p53 via ferroptosis induction in cells (Ou et al., 2016). Their subsequent work highlighted a p53-regulated ferroptosis pathway distinct from the traditional GPX4-centered model (Abrams et al., 2016). Further research led to the discovery of FINO₂ (Shimada et al., 2016) and FIN56 (Yang et al., 2016) as ferroptosis-inducing agents in 2016. Acyl-CoA synthetase long-chain family member 4 (ACSL4) was identified in 2017 as a crucial biomarker and necessary for PUFA production in ferroptosis (Doll et al., 2017). The year 2018 brought a deeper understanding of GPX4’s ferroptosis-inhibiting mechanism through its use of selenium (Ingold et al., 2018). Following this, research in 2019 revealed ferroptosis suppressor protein 1 (FSP1), a CoQ10 oxidoreductase, as an effective ferroptosis inhibitor via a GSH-independent pathway (Bersuker et al., 2019).

The exploration of ferroptosis regulation continues to yield significant discoveries. In 2020, Wang Fudi and Min Junxia’s team demonstrated that the drug auranofin induces ferroptosis by inhibiting thioredoxin reductase TXNRD, leading to cell membrane lipid peroxidation accumulation (Yang et al., 2020). This finding introduced the TXNRD protein as a novel player in the ferroptosis regulatory network. Building on this knowledge, research by Mao *et al.* in 2021 confirmed the existence of a defense mechanism against ferroptosis mediated by dihydroorotate dehydrogenase (DHODH) in the mitochondria (Mao et al., 2021). The year 2022 witnessed the identification of a non-canonical vitamin K cycle as a potent suppressor of ferroptosis (Mishima et al., 2022). Most recently, in 2023, Jiang Xuejun’s team utilized genome-wide CRISPR activation screening to discover MBOAT1 and MBOAT2 as novel ferroptosis inhibitors. These proteins act independently of GPX4 and function by remodeling phospholipids to suppress the ferroptosis (Liang et al., 2023). Additionally, the same year unveiled a novel ferroptosis monitoring mechanism involving FSP1. The most recent research in 2024 suggests anti-ferroptosis activity for 7-DHC, highlighting the ongoing exploration of potential therapeutic applications (Freitas et al., 2024).

### 2.2 Differences between ferroptosis and other regulatory cell death

Within the life cycle, programmed cell death is an essential and inevitable process under various conditions. Ferroptosis, a recently discovered form of regulated cell death, is characterized by a rise in intracellular ferrous iron accumulation and lipid-derived ROS (Yu et al., 2017). Compared to conventional cell death pathways like apoptosis, necroptosis, and pyroptosis, ferroptosis exhibits distinct features in morphology, biochemistry, core regulatory factors, and primary triggers (Table 1).

**TABLE 1:** Differences between ferroptosis and other regulatory cell death.

Type	Morphological features	Biochemical features	Core regulators	Proegumenal cause	References
**Ferroptosis**	**Mitochondria:** wrinkled; cristae reduced or even absent; membrane density condensed; outer membrane ruptured; dark stained **Cell membrane:** no rupture or blistering; cells rounded **Cytoplasm:** smaller in size **Nucleus:** no prominent change in size but chromosome cohesion is missing	Accumulation of ferrous iron and ROS, accumulation of lipid peroxides; decreased Cystine intake	VDAC2/3, Ras, NOX, TFR1, P53, CARS, GPX4, SLC7A11, HSPB1, NRF2	Decreased uptake of cysteine or glutamine and increased iron uptake inhibit GPX4	Xie et al. (2016)
**Apoptosis**	**Cell membrane:** blistering of the plasma membrane **Cytoplasm:** rounded; pseudopods contracted; cell size reduced **Nucleus:** divides; decreases in size; chromatin condenses **Organelles:** tightly arranged	Activation of cysteine asparaginase; DNA fragmentation; elevated calcium ion levels	Bax, Bak, Bcl-2 family proteins, p53, Fas	DNA damage; hypoxia; viral infectionsetc.	Li et al. (2020)
**Necroptosis**	**Cell membrane:** rupture of the plasma membrane **Cytoplasm:** swelling of the cell **Organelles:** swelling **Nucleus:** disintegration of nucleus; moderate chromatin condensation	Activation by decreased ATP levels; RIP1, RIP3, and MLKL activation; release of DAMP, PARP1 hyperactivation	RIP1, RIP3, MLKL	Ischemia-reperfusion; physical or chemical traumaetc.	Chen et al. (2022b)
**Pyroptosis**	**Cell membrane:** rupture of plasma membrane; blistering **Cytoplasm:** cell expanded and deformed **Organelle:** deformed **Nucleus:** solidified and contracted	Gasdermin-D cleaves and activates; releases pro-inflammatory factors	Caspase-1, Gasdermin D, IL-1β, NLRP3	Cell membrane permeability to potassium ions; anthrax-causing toxins; lysosomal damage; viral RNAetc.	Gao et al. (2022)
**Autophagy**	**Cell membrane:** no significant changes **Cytoplasm:** double-membrane autophagic vesicles form and accumulate **Nucleus:** lack of chromosome condensation	Elevated LC3-II/LC3-I ratio, increased lysosomal activity	ATG5, ATG7, Beclin-1, LC3, DRAM3, TFEB.	Use of mTOR, alginate inhibitors, etoposide, cruciferin, and toxic carrots	Tang et al. (2019)

VDAC2, Voltage Dependent Anion-selective Channel 2; VDAC3, Voltage Dependent Anion-selective Channel 3; NOX, Nicotinamide adenine dinucleotide phosphate Oxidases; CARS, cysteinyl-tRNA synthetase; SLC7A11, Solute carrier family 7 membrane 11; HSPB1, heat shock factor 1; DNA, deoxyribonucleic acid; BAX, BCL2-Associated X; ATP, Adenosine triphosphate; RIP1, receptor-interacting protein 1; RIP3, receptor-interacting protein 3; MLKL, mixed-lineage kinase domain-like; DAMP, damage-associated molecular pattern; PARP1, poly ADP-ribose polymerase-1; NLRP3, NOD-like receptor family pyrin domain containing 3; ATG5, Autophagy Related 5; ATG7, Autophagy Related 7; LC3, microtubule-associated proteins light chain 3; DRAM3, DRAM-Related Member 3; TFEB, Transcription factor EB.

### 2.3 Ferroptosis regulatory mechanism

**FIGURE 2:**
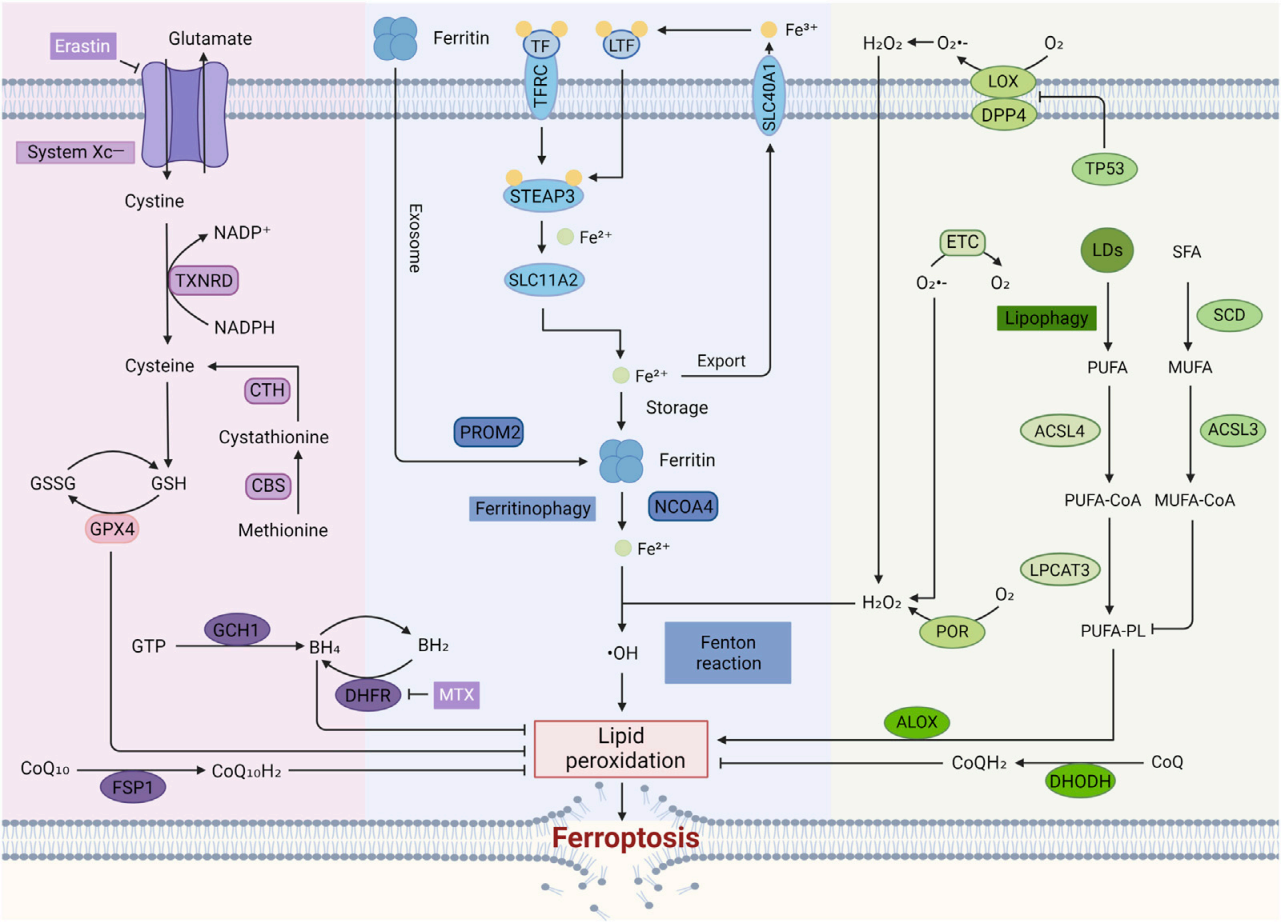
The regulatory mechanisms of ferroptosis. In the typical Ferroptosis Pathway, cystine enters the cell through System Xc^⁻^ and is converted to cysteine, leading to the production of GSH. GSH serves as a cofactor for GPX4, which plays a crucial role in converting harmful lipid peroxides into harmless lipid alcohols, thus preventing the accumulation of ROS and inhibiting ferroptosis. The accumulation of free iron in cells can trigger ferroptosis by enhancing ROS production through the Fenton reaction. Lipid peroxidation, a key characteristic of ferroptosis, is a reaction driven by free radicals that mainly affects unsaturated fatty acids in cell membranes. Additionally, enzymes like LOX and POR are also involved in this process.

Mechanistically, ferroptosis is a form of cell death triggered by lipid peroxidation of iron-dependent PUFAs with high abundance in the cell membrane (Figure 2). While the upstream signaling pathways can vary, they often converge on reducing the cell’s antioxidant capacity, either directly or indirectly affecting the activity of glutathione peroxidases (GPXs). This ultimately leads to a rise in lipid peroxidation, culminating in ferroptosis (Hirschhorn and Stockwell, 2019) with the accumulation of ROS. Specifically, the mechanism of ferroptosis mainly has the following pathways.

#### 2.3.1 Imbalance of amino acid antioxidant system

This pathway represents the canonical GPX4-regulated mechanism of ferroptosis mediated by the GSH/GPX4 axis (Seibt et al., 2019). System Xc^−^, a heterodimeric antiporter composed of SLC7A11 and SLC3A2 subunits, is a crucial antioxidant system widely distributed within the phospholipid bilayer (Koppula et al., 2021a). Cystine and glutamate are exchanged in a 1:1 stoichiometry through System Xc^−^. Cystine entering the cell is converted to cysteine by the TXNRD-mediated NADPH/NADP⁺ reaction. An alternative pathway for cysteine synthesis exists; cystathionine β-synthase (CBS) catalyzes the formation of cystathionine from methionine, an essential sulfur-containing amino acid, via the transsulfuration pathway. Cystathionine can then be further converted to cysteine, a vital component for GSH synthesis (Floros et al., 2022). Reduced GSH is oxidized to glutathione disulfide (GSSG) during its antioxidant function. Glutathione reductase (GR) then reduces GSSG back to GSH, completing the cycle (Koppula et al., 2021a). The small molecule compound Erastin can inhibit GPX4 activity by inhibiting System Xc^−^, decreasing the cell’s antioxidant capacity, which in turn increases lipid ROS (L-ROS) and ultimately leads to ferroptosis.

Recent studies highlight the involvement of TXNRD1 in ferroptosis regulation (Zhang et al., 2023). TXNRD1 plays a critical role in maintaining the non-GSH reduction pool, which is essential for utilizing and recycling small thiol-containing compounds (Mandal et al., 2010). *In vitro* studies demonstrate that TXNRD1 deficiency sensitizes chronic myeloid leukemia (CML) cells to ferroptosis induced by cysteine depletion. Furthermore, inhibition of TXNRD activity by high-dose auranofin (AUR) or the specific TXNRD1 inhibitor TRi-1 triggers ferroptosis and lipid peroxidation, which can be blocked by the ferroptosis inhibitor ferroinhibitor-1, solidifying the role of TXNRD1 in this process (Yang et al., 2020). Additionally, *Piper nigrum* L. (Piperaceae), a known TXNRD1 inhibitor derived from long pepper, significantly enhances Erastin-induced lipid peroxidation in cancer cells, suggesting the potential of targeting TXNRD1 to promote ferroptosis as a therapeutic strategy (Yang Y. et al., 2022). Interestingly, deferoxamine (DFO) protects hippocampal neurons from ferroptosis and inflammation caused by S-adenosylhomocysteine (SAH) by activating the Nrf2/TXNRD1 pathway, indicating a complex interplay between TXNRD1 and ferroptosis regulation (Hu et al., 2024). While these findings shed light on TXNRD1’s role in ferroptosis, further detailed exploration is warranted due to the recent nature of this discovery.

#### 2.3.2 Regulatory pathways independent of the GPX4 pathway

Distinct from the GPX4-centered regulatory pathway, the discovered FSP1/CoQ10, DHODH, and GCH1/BH_4_/DHFR pathway exerts its ferroptosis-inhibiting effects in a GPX4-independent manner as follows.

##### 2.3.2.1 NAD(P)H/FSP1/CoQ10 pathway

Ubiquinone (CoQ10), a lipophilic molecule primarily localized in the inner mitochondrial membrane, plays a crucial role in FSP1-mediated ferroptosis inhibition (Li W. et al., 2023). The reduced form of CoQ10, ubiquinol (CoQH_2_), acts as a potent lipophilic antioxidant by directly trapping free radicals and preventing lipid peroxidation. Additionally, CoQH_2_ indirectly regenerates another vital antioxidant, α-tocopherol, further enhancing the cellular defense against ferroptosis. Notably, α-tocopherol itself exhibits free radical scavenging activity, thereby inhibiting ferroptosis.

Mechanistically, myristoylation facilitates the recruitment of FSP1 to the plasma membrane, where it functions as an oxidoreductase. In this role, FSP1 catalyzes the reduction of CoQ10 to its antioxidant form, CoQH_2_. Studies have shown that FSP1 can also directly regenerate α-tocopherol *in vitro* (Li W. et al., 2023). Therefore, dysregulation of the FSP1-CoQ10 pathway disrupts this antioxidant defense system, rendering cells susceptible to ferroptosis.

##### 2.3.2.2 DHODH pathway

DHODH acts as a mitochondrial gatekeeper against ferroptosis by inhibiting lipid peroxidation in a CoQ-dependent manner. Located on the inner mitochondrial membrane, DHODH reduces CoQ to its antioxidant form, CoQH₂, which directly combats lipid peroxidation and prevents ferroptosis (Mao et al., 2021).

##### 2.3.2.3 GCH1/BH4/DHFR pathway

Guanylate 5′-triphosphate cyclohydrolase I (GCH1), the rate-limiting enzyme in BH_4_ biosynthesis, is pivotal in regulating ferroptosis susceptibility (Kraft et al., 2020). Cellular resistance to ferroptosis is largely determined by GCH1 expression levels. Overexpression of GCH1 selectively increases BH_4_ synthesis, leading to a reduction in ROS production. Conversely, genetic or pharmacological inhibition of GCH1 activity results in BH_4_ deficiency, contributing to the accumulation of cellular peroxides and ultimately triggering ferroptosis (Kraft et al., 2020).

Furthermore, BH_4_ participates in a redox cycle with dihydrobiopterin (BH_2_) to neutralize endogenous oxidizing free radicals. This, in turn, protects lipid membranes from peroxidation, thereby inhibiting ferroptosis. BH_4_ is regenerated from BH_2_ through a reaction catalyzed by DHFR (Kwak et al., 2011).

#### 2.3.3 Lipid metabolism pathway

A critical feature of ferroptosis is iron-dependent lipid peroxidation of polyunsaturated fatty acid-containing phospholipids (PUFA-PLs) in cell membranes. When the peroxidation rate surpasses the capacity of the cell’s ferroptosis defense system, excessive lipid peroxides accumulate, ultimately leading to membrane rupture and ferroptosis. ACSL4 and LPCAT3 play key roles in PUFA-PL synthesis, thereby promoting ferroptosis. ACSL4 catalyzes the esterification of free polyunsaturated fatty acids to coenzyme A (CoA), forming PUFA-CoA (Doll et al., 2017). Subsequently, LPCAT3 incorporates PUFA-CoA back into phospholipids (PLs) to generate PUFA-PLs (Kagan et al., 2017). Conversely, another study demonstrated that exogenous monounsaturated fatty acids (MUFAs) effectively inhibit ferroptosis by reducing oxidizable PUFA-PLs. This process involves the activation of acyl-CoA synthetase long-chain family member 3 (ACSL3), which converts MUFAs into MUFA-CoA (Magtanong et al., 2019).

Cytochrome P450 oxidoreductase (POR) indirectly contributes to lipid peroxidation by generating hydrogen peroxide (Koppula et al., 2021b). Arachidonic acid lipoxygenase (ALOX) enzymes further promote lipid peroxidation through their enzymatic reactions (Yang et al., 2016).

The tumor suppressor protein p53 (TP53) acts as a negative regulator of ferroptosis through a transcriptionally independent mechanism by inhibiting the activity of dipeptidyl peptidase-4 (DPP4) (Xie et al., 2017). In cells lacking p53, DPP4 fails to accumulate in the nucleus, leading to its increased presence at the plasma membrane. This membrane-associated DPP4 then promotes lipid peroxidation, ultimately triggering ferroptosis.

Mitochondria are crucial organelles, not only for regulating intracellular energy homeostasis but also for signaling cell death pathways (Zorov et al., 2014). Within the mitochondria, incomplete electron transfer during the electron transport chain (ETC.) can lead to the formation of superoxide radicals (O₂⁻) through oxygen reduction. These superoxide radicals are rapidly converted to H₂O₂ by the enzyme superoxide dismutase (SOD). However, excessive or dysregulated H₂O₂ production can contribute to the formation of lipid peroxides, ultimately promoting ferroptosis (Stockwell and Jiang, 2020).

#### 2.3.4 Iron metabolism pathways

Ferroptosis is critically dependent on the Fenton reaction, where Fe^2^⁺ reacts with H₂O₂ to generate highly reactive hydroxyl radicals (OH•). These hydroxyl radicals trigger the peroxidation of lipids within the cell membrane, ultimately leading to ferroptosis (Henning et al., 2022). Various methods that increase the intracellular levels of free iron can promote ferroptosis through this mechanism. Iron metabolism is a tightly regulated process involving multiple steps. Dietary non-heme iron exists primarily as insoluble Fe³⁺, which needs to be converted to the more readily absorbed Fe^2^⁺ for cellular uptake. Transferrin (TF) in the serum serves as the primary carrier for Fe³⁺, delivering it to transferrin receptors (TFRC) located on the cell membrane (Lambe et al., 2009). Lactotransferrin (LTF), similar to TF, can also promote ferroptosis by enhancing cellular iron uptake (Wang Y. et al., 2020). After internalization, STEAP3 metalloreductase facilitates the reduction of Fe³⁺ to Fe^2^⁺ within endosomes. Subsequently, iron efflux proteins like solute carrier family 40 member 1 (SLC40A1) transport excess iron out of the cell, where it is re-oxidized back to Fe³⁺ in the extracellular space. Additionally, solute carrier family 11 member 2 (SLC11A2) mediates the release of Fe^2^⁺ from endosomes into the cytoplasm, further contributing to increased free iron levels and promoting ferroptosis (Tang et al., 2021).

### 2.4 Ferroptosis inducer and inhibitors

Ferroptosis, a recently discovered form of regulated cell death, is characterized by the iron-dependent accumulation of toxic lipid peroxides within the cell membrane (Wang Y. et al., 2022). By targeting key players in the ferroptosis signaling pathways, researchers have successfully developed a diverse range of ferroptosis inducers and inhibitors, as summarized in Table 2. This ongoing development holds significant promise for advancing our understanding of the ferroptosis mechanism and its potential as a therapeutic target. The exploration of ferroptosis inducers and inhibitors has the potential to unlock new breakthroughs in treating various diseases.

**TABLE 2:** Common inducers and inhibitors of ferroptosis.

Types	Mechanism	Small molecules	References
Inducer	Inhibition System Xc^-^	erastin, sorafenib, sulfasalazine, glutamate, buthionine	Li et al. (2020), Xie et al. (2016), Chen et al. (2021d), Rochette et al. (2022)
	Suppression GPX4	RSL3, FIN56, ML210, ML162, altretamine, withaferinA, FINO2, DPI family members, sorafenib	Chen et al. (2021d), Du and Guo (2022), Lei et al. (2022)
	Suppression SLC7A11	BAPI, P53	Lei et al. (2022)
Inhibitor	Iron chelators	CPX, CPX-O, DFO, DFP, DFX	Du and Guo (2022)
	Inhibits GPX4 degradation	CDDO, Dopamine	Du and Guo (2022)
	Reduces lipid peroxidation	Vitamin E, trolox, tocotrienols, BHT, BHA, CoQ10, idebenone; XJB-5-131, deferoxamine, cyclipirox, deferiprone; CDC, baicalein, PD-146176, AA-861, zileuton; vildagliptin, alogliptin, andlinagliptin, VK Full Restore modeVKH2, SRS15-72B, SRS15-72A, SRS16-80, SRS16-86, SRS11-9	Rochette et al. (2022)

#### 2.4.1 Inhibition of System Xc^−^ induced ferroptosis

System Xc^−^ is a heterodimeric amino acid antiporter embedded in the plasma membrane and composed of two subunits: the light chain SLC7A11 and the heavy chain SLC3A2 (Kim et al., 2021). It facilitates the antiparallel exchange of cystine and glutamate at a 1:1 ratio across the phospholipid bilayer. Intracellular cystine is then reduced to cysteine, a vital precursor for GSH synthesis. GSH, in turn, serves as a cofactor for GPX4, a key antioxidant enzyme that protects cells from lipid peroxidation damage (Tang et al., 2016). Therefore, inhibition of System Xc^−^ activity disrupts cystine import, consequently limiting GSH synthesis and reducing GPX4 activity. This decline in cellular antioxidant capacity leads to ferroptosis (Gao et al., 2022). Erastin, for example, is known to induce ferroptosis by promoting the accumulation of lipid-ROS and compromising the cell’s overall antioxidant defense system. The tumor suppressor protein p53 plays a distinct role in ferroptosis regulation compared to inducers like Erastin. While p53 mutations are prevalent in many cancers, these mutations can promote ferroptosis (Guo et al., 2021). Specifically, acetylation-deficient p53 mutants can inhibit cystine uptake via System Xc^−^, ultimately leading to ferroptosis induction.

#### 2.4.2 Inhibiting GPX4-induced ferroptosis

GPX4 is a selenium-dependent enzyme that functions as a crucial antioxidant by inhibiting lipid peroxidation, a process that damages cell membranes (Beharier et al., 2020). GPX4 utilizes its catalytic activity to reduce the toxicity of lipid peroxides, thereby maintaining the stability and integrity of the phospholipid bilayer (Zhang W. et al., 2022). Under normal conditions with sufficient GSH levels, GPX4 can efficiently convert lipid peroxides back to their non-toxic phospholipid counterparts using GSH as a substrate. Consequently, inhibiting GPX4 activity disrupts intracellular redox homeostasis, leading to oxidative stress. RSL3 is a well-characterized inhibitor of GPX4. It acts by covalently binding to GPX4, inactivating the enzyme and preventing its essential function in reducing lipid peroxides (Li D. et al., 2022). Notably, GSH, not superoxide, serves as the direct substrate for GPX4, facilitating the reduction of lipid peroxides, not superoxide radicals. Cysteine deficiency can indirectly contribute to GPX4 inactivation. Since GSH synthesis requires cysteine as a precursor, a lack of cysteine would limit GSH production, thereby hindering GPX4’s ability to function properly. FIN56, a specific ferroptosis inducer, exploits this vulnerability by promoting GPX4 degradation, ultimately leading to ferroptosis induction.

#### 2.4.3 Inhibiting SLC7A11-induced ferroptosis

SLC7A11, a key component of System Xc^−^, is a specific heterodimeric antiporter protein on the plasma membrane. This protein mediates the antiparallel exchange of cystine and glutamate, playing a vital role in amino acid transport (Yu et al., 2020). SLC7A11 is a crucial regulator of ferroptosis, a form of regulated cell death dependent on iron. Under normal conditions, SLC7A11 facilitates cystine import, which is converted to cysteine, a precursor for GSH synthesis. GSH, in turn, serves as a cofactor for GPX4, a vital antioxidant enzyme that protects cells from lipid peroxidation damage (Zhao et al., 2021). Therefore, downregulation of SLC7A11 expression disrupts cystine import, consequently inhibiting the cysteine-GSH-GPX4 pathway. This ultimately leads to a decline in cellular antioxidant capacity and the accumulation of lipid peroxides, triggering ferroptosis (Feng et al., 2022). Besides, p53, a tumor suppressor protein, can regulate ferroptosis by directly inhibiting SLC7A11 expression. Importantly, p53 achieves this through two main mechanisms: 1) directly binding to the SLC7A11 promoter region, and 2) interacting with ubiquitin-specific processing protease 7 (USP7) to reduce histone H2B monoubiquitination on the SLC7A11 promoter. Both mechanisms lead to decreased SLC7A11 transcription and reduced cystine import, ultimately promoting ferroptosis.

#### 2.4.4 Iron chelators inhibit ferroptosis

Iron chelators offer another strategy for inhibiting ferroptosis. Deferoxamine, a commonly used chelator, is a polyamide capable of sequestering iron ions (Wu et al., 2022). Ciclopirox, another effective iron chelator, is a highly permeable polyamide with similar iron-binding properties.

#### 2.4.5 Inhibiting GPX4 degradation inhibits ferroptosis

GPX4 can degrade small molecule peroxides and certain lipid peroxides, inhibiting lipid peroxidation. GPX4 specifically converts lipid hydroperoxides (LOOH) into their corresponding alcohols (LOH). When GPX4 activity declines, these lipid peroxides accumulate. Fe^2^⁺ ions can then readily oxidize these accumulated lipids, generating a large amount of ROS and, ultimately, ferroptosis (Li B. et al., 2022). GPX4 also plays a role in maintaining intracellular redox homeostasis by scavenging H₂O₂ within the cell. Consequently, increasing GPX4 expression can reduce the concentration of intracellular ROS and inhibit ferroptosis. However, chaperone-mediated autophagy, a cellular degradation process, can target GPX4 for breakdown (Elakkad et al., 2021). 2-Amino-5-chloro-N,3-dimethylbenzamide (bardoxolone, CDDO) is a triterpenoid compound that has been shown to inhibit the degradation of GPX4 by targeting heat shock protein 90 (HSP90), a molecular chaperone involved in protein folding and degradation. By protecting GPX4, CDDO can potentially protect cells from ferroptosis (Wu et al., 2019).

#### 2.4.6 Mitigation of lipid peroxidation and inhibition of ferroptosis

Ferroptosis, a form of regulated cell death, is characterized by two key events: ROS production and lipid peroxidation. Lipid peroxidation is a process where oxidants, such as free radicals or non-radical species, attack PUFAs within cell membranes (Dong et al., 2021). These fatty acids, with their carbon-carbon double bonds, are crucial for maintaining cell structure and function, making them prime targets for ROS attack. The peroxidation of these lipids leads to the formation of lipid peroxides, which play a key role in the execution of ferroptosis. Fortunately, antioxidant molecules can help prevent ferroptosis by scavenging ROS and inhibiting lipid peroxidation. The VKH series compound, specifically 2VK in its fully reduced form, demonstrates the ability to inhibit lipid oxidation and, consequently, has the potential to function as a ferroptosis inhibitor.

### 2.5 Detection method for ferroptosis

**FIGURE 3:**
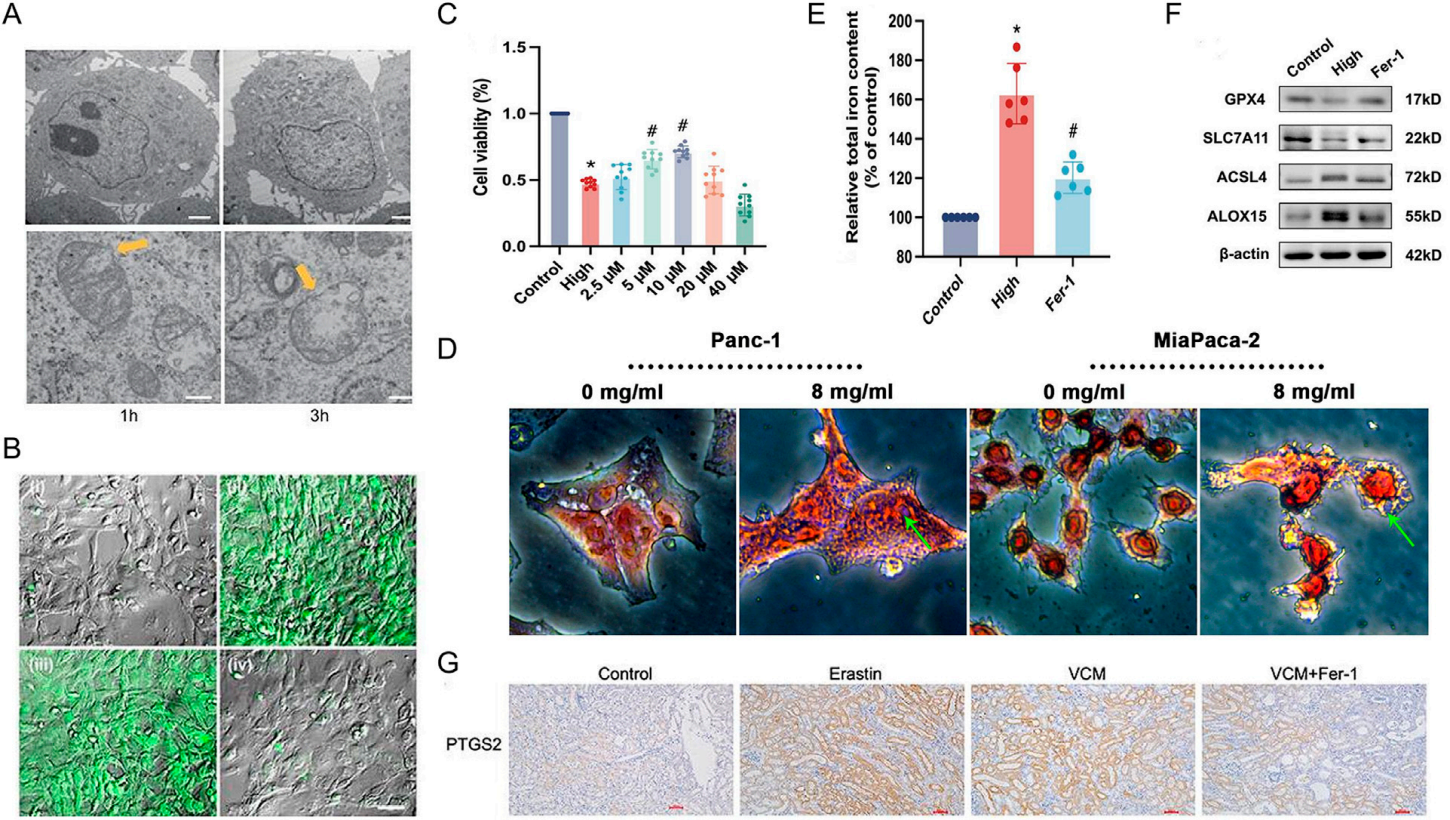
Ferroptosis detection method. **(A)** Electron micrographs of mouse embryonic fibroblasts (MEF) showing a time-dependent outer mitochondrial membrane rupture (yellow arrows) upon ferroptosis induction using RSL3 (50 nM; scale bars 2 μm top row, 200 nm bottom row) while nuclear integrity is preserved (Lewerenz et al., 2018). Copyright ^©^ 2018 January Lewerenz, *et al.* **(B)** Live cell imaging in ARPE19 cells loaded with the fluorescent indicator, Liperfluo, which recognizes lipid peroxides that are markers for ferroptosis activation, showed higher liperfluo emission intensity (green) in RSL3 (3 μM for 12 h, positive control) (Gupta et al., 2023). Copyright ^©^ 2022 Urvi Gupta, *et al.* **(C)** Ferrostatin-1 (Fer-1) alleviates ferroptosis in corpus cavernosum smooth muscle cells (CCSMCs) the results of the Cell Counting Kit-8 (CCK-8) assay (Xu et al., 2023). Copyright ^©^ 2022 Wenchao Xu, *et al.* **(D)** Compared with the control group, Prussian blue staining showed heavy cellular iron accumulation in the Huaier group (Zhu et al., 2022). Copyright ^©^ 2022 Zeen Zhu, *et al.* **(E)** Ferrostatin-1 (Fer-1) alleviates ferroptosis in corpus cavernosum smooth muscle cells (CCSMCs relative total iron content, n = 6 for each group) (Xu et al., 2023). Copyright ^©^ 2022 Wenchao Xu, *et al.* **(F)** Ferrostatin-1 (Fer-1) alleviates ferroptosis in corpus cavernosum smooth muscle cells (CCSMCs) representative Western blot results for GPX4, SLC7A11, ACSL4, and ALOX15 (Xu et al., 2023). Copyright ^©^ 2022 Wenchao Xu, *et al.* **(G)** IHC staining showed that GPX4 and SLC7A11 were downregulated and PTGS2 was upregulated in the kidney tissues of VCM-treated mice. In addition, Fer-1 restored the expressions of GPX4 and SLC7A11 and reduced the expression of PTGS2 in mice injected with VCM (Yin et al., 2023). Copyright ^©^ 2023 Xuedong Yin, *et al.*

Ferroptosis is a regulated cell death process driven by iron-dependent accumulation of lipid peroxides. Key factors promoting ferroptosis include increased iron levels, free radical production, availability of polyunsaturated fatty acids within cells, and elevated lipid peroxidation. Due to the distinct morphological, biochemical, and genetic hallmarks of ferroptosis, several methods are currently employed for its detection. These methods include assessing mitochondrial morphology and function, lipid peroxidation, cell viability, iron metabolism levels, and the expression of ferroptosis marker genes and proteins (Figure 3).

#### 2.5.1 Mitochondrial morphology and function detection

Electron microscopy is a powerful tool for assessing mitochondrial morphology, a hallmark of ferroptosis. It allows visualization of various ferroptosis-induced changes, including mitochondrial volume reduction, increased outer membrane density, outer membrane rupture, cristae damage, shrinkage, increased electron density, and vacuolization (Figure 3A) (Zhou et al., 2022). Electron microscopy can be employed to visualize these morphological changes, aiding in determining whether cells are undergoing ferroptosis. Mitochondrial membrane potential (ΔΨm) is a valuable indicator of ferroptosis. The cationic dye JC-1 accumulates in the mitochondria with high ΔΨm, forming red fluorescent polymers that emit light at 590 nm when excited at 488 nm (Zhang et al., 2020). Conversely, in cells with low ΔΨm, JC-1 remains as green fluorescent monomers, emitting light at 527 nm upon excitation at 488 nm (Yang W. et al., 2022). Flow cytometry can measure the fluorescence intensity of JC-1, providing a quantitative assessment of ΔΨm and potentially indicating ferroptosis.

#### 2.5.2 Lipid peroxidation detection

ROS and, specifically, lipid ROS play a crucial role in ferroptosis. Iron accumulation within cells disrupts the antioxidant system, potentially leading to the direct generation of excessive ROS via the Fenton reaction. This, in turn, contributes to increased oxidative damage (Kuo et al., 2014). Therefore, the detection of ROS levels serves as another common method for ferroptosis assessment. Flow cytometry with the C11-BODIPY fluorescent probe is a valuable tool for detecting intracellular and lipid ROS(74). C11-BODIPY 581/591 is a lipophilic dye that accumulates in the cell membrane. When excited at 488 nm or 568 nm, the non-oxidized probe emits red fluorescence at 590 nm (Petronek et al., 2022). However, upon oxidation, the emitted light shifts to green fluorescence at 510 nm (Lai et al., 2013). The intensity of the green fluorescence signal, measured by flow cytometry (Figure 3B), is positively correlated with the level of ferroptosis.

#### 2.5.3 Cell viability detection

Ferroptosis ultimately leads to cell death, allowing for detection methods like MTT and CCK-8 assays. The MTT assay relies on the ability of viable cells with functioning mitochondrial succinate dehydrogenase to convert a yellow MTT reagent to a blue formazan product. Dead cells lack this activity. By dissolving the formazan crystals and measuring their light absorbance at 570 nm, the MTT assay indirectly reflects the number of viable cells (Chen L. et al., 2021). Similarly, the CCK-8 assay employs a tetrazolium compound (WST-8) that is reduced by mitochondrial dehydrogenases in living cells to a yellow formazan product. The amount of formazan formation, measured by absorbance at 450 nm, is proportional to cell viability. While both assays can identify ferroptosis-induced cell death, it should be borne in mind that they cannot distinguish ferroptosis from other cell death processes like apoptosis or necrosis (Figure 3C). Therefore, these methods should be used with other ferroptosis-specific markers for a more definitive diagnosis.

Ferroptosis disrupts the fine structure of cells, causing mitochondrial shrinkage compared to healthy cells. Additionally, ferroptosis can lead to the reduction or disappearance of mitochondrial cristae, outer membrane rupture, and vacuolization (Ma et al., 2020). These morphological changes can be visualized using various staining methods for ferroptosis detection. Examples include calcein-AM staining, which assesses cell viability, and Prussian blue staining, which detects iron accumulation, a hallmark of ferroptosis (Figure 3D). However, methods like trypan blue staining, which generally detect compromised cell membranes, might not be ferroptosis-specific and could identify other forms of cell death.

#### 2.5.4 Iron metabolism level detection

Iron metabolism plays a critical role in ferroptosis susceptibility. Iron functions in various cellular processes, including lipid peroxidation and redox reactions. The absorption, transport, storage, and utilization of iron ions all influence a cell’s vulnerability to ferroptosis. In the bloodstream, Fe³⁺ binds to transferrin for transport. Upon entering cells via transferrin receptors, Fe³⁺ is reduced to Fe^2^⁺ and released into the labile iron pool (LIP) in the cytoplasm. Excess iron is stored in ferritin for later use (Wang YM. et al., 2022). The LIP primarily consists of Fe^2^⁺, a highly reactive form that can participate in the Fenton reaction. This reaction generates hydroxyl radicals, which readily attack polyunsaturated fatty acids within cell membranes, leading to the formation of a large amount of lipid peroxides (ROS) and, ultimately, ferroptosis-induced cell death (He F. et al., 2022). Therefore, the cellular iron content, which is often elevated in ferroptosis, can serve as an indicator of this cell death process (Figure 3E). However, iron levels alone cannot definitively diagnose ferroptosis.

#### 2.5.5 Expression detection of ferroptosis marker-related genes and proteins

Ferroptosis can be triggered by inhibiting cellular cystine import, a vital precursor for GSH synthesis. This inhibition, caused by agents like Erastin, depletes intracellular GSH stores. Consequently, the activity of the antioxidant enzyme GPX4 is compromised. This leads to the accumulation of lipid peroxides, which, when exceeding a critical threshold, triggers ferroptosis (Pontel et al., 2022). Similarly, direct inhibition of GPX4 activity with compounds like RSL3 can induce ferroptosis. The expression levels of ferroptosis marker genes, including *PTGS2*, *NOX1*, *FTH1*, *COX2*, *GPX4*, and *ACSL4*, can be assessed using real-time quantitative PCR (qPCR). Additionally, Western blotting (Figure 3F), immunohistochemistry (Figure 3G), and immunofluorescence staining can be employed to detect the protein levels of these markers, aiding in determining whether cells have undergone ferroptosis.

## 3 Overview of natural products

### 3.1 Overview of natural products

Natural products and their derivatives have emerged as promising therapeutic candidates for various diseases, including diabetes, cancer, obesity, and neurological disorders (Kim et al., 2018; Yang et al., 2018; Luo et al., 2018). These natural compounds hold significant value in traditional medicine and modern drug discovery due to their remarkable structural diversity and biological activity. Natural products typically comprise complex molecules built upon a carbon skeleton. This carbon backbone forms chemical bonds with elements like hydrogen, oxygen, nitrogen, and sulfur. The specific arrangement and connection of these atomic groups dictate the unique properties and functionalities of each natural product (Hu et al., 2021).

In living organisms, the intricate biosynthesis of natural products is primarily orchestrated by enzymes. These specialized biocatalysts selectively accelerate specific reactions under defined cellular conditions, enabling the efficient execution of complex chemical syntheses. Therefore, natural products often exhibit distinct stereochemical features, such as chiral centers and specific double-bond configurations. Notably, the structure of a natural product is frequently intertwined with its biological activity. Many pharmaceuticals and bioactive molecules are derived or inspired by natural products, and their specific structures dictate their interactions with cellular molecules within an organism. By thoroughly elucidating the structures of natural products, we can unveil the mechanisms underlying their pharmacological effects, thereby providing a valuable foundation for novel drug design and synthesis.

Natural products exhibit a diverse range of biological activities, including antibacterial, antioxidant, anti-inflammatory, antitumor, and antiviral properties. Antibacterial activity can inhibit the growth and reproduction of bacteria, offering protection against infections. Antioxidant activity neutralizes free radicals, shielding cells from oxidative damage (van der Valk et al., 2004). Anti-inflammatory activity helps reduce inflammation and alleviate associated disease symptoms. Antitumor activity disrupts the growth and metastasis of cancer cells. Additionally, some natural products demonstrate antiviral activity, hindering viral replication and spread.

Therefore, natural products represent a treasure trove for drug discovery and development, offering immense potential for novel therapeutics. However, their translation into clinical applications necessitates further investigation to elucidate their pharmacological mechanisms and ensure safety. This includes comprehensively evaluating drug stability, bioavailability, and potential side effects (de Sá Coutinho et al., 2020). Natural product databases and targeted development strategies serve as invaluable tools for guiding and expediting research in this field, ultimately paving the way for the discovery and development of new drugs.

### 3.2 Natural product database

Natural product databases are centralized resource libraries that store and organize information on natural products. These databases encompass a wealth of data on the chemical composition and biological activities of natural organisms, including plants, animals, and microorganisms. Additionally, they provide insights into the action targets of these natural products against various diseases. This comprehensive information serves as a crucial foundation for scientific research and industrial applications of natural products. In Table 3, we have compiled and organized relevant information from current natural product databases. Indeed, this curated resource will be a valuable tool for researchers in scientific research and drug development, ultimately accelerating drug discovery, improving disease treatment, and propelling advancements in scientific inquiry.

**TABLE 3:** Common natural products database.

Database	Data source	Advantage	Disadvantage	Web link
Dictionary of Natural Products	Natural product	The only comprehensive, fully edited natural products database with full support for structure searches	—	http://www.chemnetbase.com/
COCONUT	Natural product	Stereochemical forms, literature, biological sources, geographical locations and various pre-calculated molecular properties	—	coconut.naturalproducts.net/
Super Natural 3.0	Natural compounds	Big and comprehensive	—	bioinf-applied.charite.de/supernatural_3/index.php?site=home
MPOD	Plant	MPOD provides functions such as gene homologous gene candidate query, homology comparison of gene families in different species, metabolite distribution and gene expression correlation analysisetc.	—	http://medicinalplants.ynau.edu.cn/
CMAUP	Plant	The relationship between targets, pathways and diseases is relatively clear	—	bidd.group/CMAUP/index.html
Alkamid	Plant	Specific ethnopharmacological data can be directly searched, along with their physicochemical properties and botanical origins	The amount of data collected on compounds is small	alkamid.ugent.be/
AfroDb	Africa medicinal plant	Covers the entire African continent classified by “drug-like”, “lead-like” and “fragment-like” subgroups	It has been a long time since the last update, so the timeliness is low	zinc.docking.org/catalogs/afronp/
ETM-DB	Ethiopian herbal medicine	Contains Ethiopian herbs, related compounds and target genes/proteins	The content is relatively unique and only contains Ethiopian herbal related compounds	biosoft.kaist.ac.kr/etm/home.php/
NuBBE	Brazilian plants	Group drug similarity assessment compounds based on source of acquisition	There are no functions or diseases associated with these compounds	www.cas.org/zh-hans/resources/gated-content/case-studies/biodiversity
CEMTDD	Chinese ethnic minority herbal medicine	The relationship between compounds in Chinese medicinal materials and their targets	Diseases cannot be queried as search keywords	http://www.cemtdd.com/
CHDD	Chinese herbal medicine	Detailed records of the properties, functions, dosages and other details of herbal medicines	—	https://www.meandqi.com/herb-database
TCMDB@Taiwan	Traditional Chinese medicine	Has the largest data source of traditional Chinese medicine	Show only relationships between herbs, ingredients and compounds	http://tcm.cmu.edu.tw/
ETCM	Traditional Chinese medicine	Provides predicted target genes for traditional Chinese medicine ingredients, herbs and formulas	—	http://www.tcmip.cn/ETCM/
TCMID	Traditional Chinese medicine	Database about traditional Chinese medicine, including information on herbal ingredients, drugs and diseasesetc.	—	https://bidd.group/TCMID/index.html
TCMSP	Traditional Chinese medicine	The unique systems pharmacology platform of Chinese herbal medicine can capture the relationship between drugs, targets and diseases	—	https://tcmsp-e.com/tcmsp.php
HIT	Traditional Chinese medicine	Mainly studies herbal ingredients and can directly obtain relevant protein target information	Except for key features, the rest of the information is less complete	http://www.badd-cao.net:2345/
ChEMBL	Bioactive molecules	Database of bioactive molecules with drug-like properties, bringing together chemical, bioactive and genomic data	—	http://www.ebi.ac.uk/chembl/
TTD	Drug data	Sources of data on target interactions compared with successful drugs	Lack of target interaction	db.idrblab.net/ttd/
PAMDB	Microbial natural products	Contains extensive metabolomic data and metabolic pathway maps on *Pseudomonas aeruginosa*	—	*pseudomonas*.umaryland.edu/PAMDB.htm
NP Altas	Microbial natural products	Natural products of microbial origin in primary scientific literature. This includes bacterial, fungal and cyanobacterial compounds that have been documented	Compounds from plants, invertebrates or other higher organisms are not included	www.npatlas.org
ProCarDB	Prokaryotic carotenoids	Provides information on the pathways, enzymes, coding genes, and bacterial species that possess carotenoids, *etc.*, for the synthesis of carotenoids	Only looking for content related to carotenoids	bioinfo.imtech.res.in/servers/procardb/
Seaweed Metabolite Database	Metabolites of seaweed	The geographical origin, extraction and biological activities of seaweed secondary metabolites are provided. In addition to structural and attribute information	—	http://swmd.co.in/index.php
MarinLit	Marine natural products	A database dedicated to the study of marine natural products with powerful anti-replication capabilities	—	http://pubs.rsc.org/marinlit/
MNPD	Marine natural products	Provides information on various physicochemical and pharmacokinetic properties, standardized biological activity data, systematic classification and geographical distribution of source organisms, and relevant literature citations	—	https://www.cmnpd.org/

Natural product databases are pivotal in the realms of biodiversity research and pharmaceutical innovation. Despite their repository of substantial valuable data, they confront numerous challenges. The prevalence of data incompleteness is a critical concern, given the vastness of global biodiversity that remains undocumented. The databases' data refresh rate frequently lags behind the swift progression of scientific inquiry, leading to outdated information. The lack of standardization across databases further complicates the integration and comparative analysis of data. Limitations in access and utilization, variability in data quality, the intricacies of technological integration, and the complexities of intellectual property and ethical considerations are hurdles that must be surmounted in the evolution of natural product databases. To optimize their utility, fostering international collaboration, promoting data sharing, enforcing data standardization, enhancing the quality and velocity of data updates, and addressing the ethical and legal dimensions of data usage are imperative.

## 4 Effect of natural products regulating ferroptosis on diabetic complications

### 4.1 Overview of diabetes complications

Diabetes mellitus extends beyond simply elevated blood sugar levels. Over time, it can lead to a cascade of complications affecting various peripheral tissues, including the kidneys, cardiovascular system, retina and nervous system. While the clinical presentation and pathological hallmarks of these complications have been extensively studied, the underlying molecular mechanisms remain elusive. This lack of complete understanding hinders the development of truly effective treatments for both diabetes itself and its associated complications. Notably, strict blood sugar control, although crucial, may not fully prevent these complications from arising. Mounting evidence suggests that dysregulation of other cellular processes, such as the production of harmful reactive metabolites, also contributes to diabetes development (Demir et al., 2021). Iron metabolism disorders can affect various complications of diabetes through iron-induced free radical damage, inflammatory responses, and mitochondrial dysfunction (Li L. et al., 2023; Ke et al., 2023). Therefore, a comprehensive approach to managing diabetes necessitates addressing not only the core hyperglycemia but also the microvascular and macrovascular complications it triggers. Microvascular complications primarily encompass DKD, DR, diabetic cardiomyopathy (DCM), and diabetic peripheral neuropathy (DPN). Macrovascular complications include coronary artery disease, stroke, and peripheral arterial disease (PAD), leading to conditions like intermittent claudication. These complications significantly compromise patients' health, reducing their quality of life and potentially leading to disability or even death. Consequently, a comprehensive understanding of the specific mechanisms underlying each diabetic complication is essential (Table 4). This knowledge will pave the way for the identification of novel therapeutic targets and the development of targeted drugs to effectively prevent or manage these debilitating conditions.

**TABLE 4:** Characteristics and mechanisms of various diabetic complications.

Diabetic complications	Complication characteristics	Pathogenesis	References
Diabetic nephropathy	Persistent proteinuria, elevated arterial blood pressure, and reduced glomerular filtration rate	Alterations in glomerular hemodynamics, oxidative stress and inflammation, as well as interstitial fibrosis and renal tubular atrophy	Alicic et al. (2017)
Diabetic cardiomyopathy	Diastolic and systolic dysfunction, left ventricular hypertrophy, myocyte hypertrophy, and fibrosis are major causes of heart failure in diabetic patients	Exposure of the heart to a hyperglycemic environment, along with elevated levels of fatty acids (FA), cytokines, and non-enzymatic advanced glycation end products (AGE), disrupts various signaling pathways. This includes decreased AMPK signaling and heightened PKC and MAPK signaling	Wang et al. (2006)
Diabetic retinopathy	Dysfunction and increased permeability of retinal capillary endothelial cells (RCECs), pericyte damage in retinal vessels, endothelial cell dysfunction, and basement membrane thickening, leading to rupture of the blood-retinal barrier	Microangiopathy leads to retinal ischemia, visual impairment, activation of the polyol pathway, accumulation of late glycosylation end products (AGEs), oxidative stress, activation of protein kinase C, inflammation, upregulation of the renin-angiotensin system and vascular endothelial growth factor (VEGF), and ultimately disruption of the blood-retinal barrier (BRB)	Cheung et al. (2010)
Diabetic peripheral neuropathy	The initial stages of the disease showed demyelination of nerve fibers and degeneration of axons, along with Schwann cell proliferation. As the disease progressed, there was further axon degeneration and disappearance of myelin fibers. Concurrently, a regenerative plexus formed during myelin fiber degeneration, followed by a decrease in density	Insulin resistance affects peripheral nerves, causing the insulin receptors of Schwann cells and axons to become unresponsive to insulin. This leads to damage through various pathways such as the polyol pathway, hexosamine pathway, activation of protein kinase C pathway, and formation of AGEs, resulting in indirect or direct damage to nerve cells and axons	Elafros et al. (2022)

### 4.2 Natural product Classes that regulate ferroptosis and affect diabetic complications

#### 4.2.1 Flavonoids

Flavonoids, a diverse group of naturally occurring polyphenolic secondary metabolites, are abundant in plants like vegetables, tea leaves, and botanical drugs. Their therapeutic potential against various diseases, including cancer, bacterial infections, organ dysfunction, and oxidative stress, has garnered significant research interest. Notably, oxidative stress is increasingly recognized as a crucial factor alongside hyperglycemia in the development of diabetic nephropathy (Cheng et al., 2016). The System Xc^−^/GPX4 pathway plays a vital role in eliminating lipid peroxides, acting as a key antioxidant defense system against oxidative stress and a regulator of ferroptosis, a form of iron-dependent cell death (Chen et al., 2021b). Upregulation of the nuclear factor E2-related factor 2 (Nrf2) pathway can modulate the expression of GPX4, SLC7A11 (encoding the light chain of System Xc^−^), ferritin heavy chain 1 (FTH-1), and transferrin receptor 1 (TFR1), thereby restoring antioxidant capacity and normalizing iron homeostasis, ultimately inhibiting ferroptosis (Li et al., 2021). *Glycyrrhiza uralensis* Fisch. ex DC. (Fabaceae) extracts, rich in compounds like glabridine (Glab), have demonstrated broad antioxidant effects in various diseases. Tan *et al.* induced DM in rats using STZ and treated them with Glab at a dosage of 50 mg/kg for 28 days. Administered intragastrically at a dose of 5 mg/kg, Rosi (rosiglitazone) served as the positive drug intervention group. The study observed a significant elevation in AGEs in the kidneys of DM rats, which was attenuated by Glab administration. Concurrently, the levels of ROS and MDA were markedly increased in the kidneys of DM rats due to AGE-induced oxidative stress. Glab treatment not only mitigated these increases but also restored the activity of antioxidant enzymes, including CAT, GSH, and SOD, which were significantly suppressed in DM rats. Furthermore, Glab was found to reduce the levels of GPX4, SLC7A11, SLC3A2, MDA, iron concentration, and TFR1 expression, thereby inhibiting ferroptosis. These findings suggest that Glab ameliorates diabetic nephropathy by enhancing antioxidant defense mechanisms and suppressing ferroptosis pathways (Tan et al., 2022). Renal tubular injury is a well-established hallmark of diabetic nephropathy (Czajka and Malik, 2016). Quercetin (QCT) has been shown to impede the progression of this condition by upregulating Nrf2. Li D. *et al.* investigated the impact of varying concentrations of QCT (6.25, 12.5, 25, 50, or 100 μM) on the viability of HK-2 cells. After a 48-h incubation period, it was observed that cell viability was notably diminished in the HG group. However, the HG-induced reduction in cell viability was significantly counteracted in all QCT-treated groups, with the most pronounced effect observed at a QCT concentration of 25 μM. Subsequent research revealed that QCT exerts its protective effect against ferroptosis in HK-2 cells by modulating the expression of key ferroptosis-related proteins. Specifically, QCT down-regulated the expression of TFR1 and upregulated the expression of GPX4, FTH-1, and SLC7A11, thereby attenuating ferroptosis. Additionally, QCT activates the Nrf2/HO-1 signaling pathway by increasing Nrf2 and HO-1 levels, thereby inhibiting ferroptosis in these cells and potentially improving diabetic nephropathy (Feng et al., 2023). Mesangial cells (GMCs) are specialized smooth muscle cells within the glomerular capillaries, and their damage constitutes a fundamental pathological change in diabetic nephropathy (Yang et al., 2021). Hou *et al.* demonstrated that in STZ-induced diabetic rats, the oral administration of Puerinda (PUR) at a dosage of 100 mg/kg/day for a period of 8 weeks significantly mitigated GMCs damage, thereby ameliorating diabetic nephropathy. Subsequent research elucidated that PUR’s protective effects were mediated through the restoration of iron metabolic homeostasis, which in turn inhibited the hyperglycemia-induced overproduction of extracellular matrix in GMCs, culminating in a reduction of cellular damage. In particular, we found that the high-concentration (10 μM) treatment group showed a better treatment effect compared to the low-concentration (1 μM) treatment group (Hou et al., 2024). This growing body of evidence suggests that flavonoids can influence the course of diabetes and its complications by regulating the ferroptosis pathway, offering a promising new therapeutic approach for this debilitating condition.

#### 4.2.2 Polyphenolics

Phenolic compounds (PCs) are a structurally diverse class of naturally occurring molecules abundant in plants. They possess various biological activities, notably antioxidant, antimicrobial, anticancer, anti-inflammatory, and neuroprotective effects. The ability of various PCs to inhibit ferroptosis stems largely from their influence on similar mechanisms, all centered around reducing oxidative stress (Lesjak et al., 2022). These mechanisms include inhibiting ROS production, iron accumulation, and the upregulation of antioxidant enzymes like GPX4 and the transcription factor Nrf2. Nrf2 plays a critical role in the body’s endogenous antioxidant defense system (Wang C-Y. et al., 2020). The Nrf2/ARE signaling pathway regulates the expression of genes involved in GPX4 synthesis and NADPH production. By enhancing these antioxidant mechanisms, the pathway helps neutralize accumulated superoxide radicals and mitigate oxidative stress. Consequently, Nrf2 activation offers potential benefits in ferroptosis, diabetic complications, and other related diseases (Wei et al., 2022). Previous studies have established that curcumin, a renowned PC, exerts its antioxidant effects by modulating the Nrf2 signaling pathway. This insight prompts the inquiry into whether curcumin could potentially regulate Nrf2 to mitigate ferroptosis-associated complications in diabetes. In alignment with this hypothesis, Zhang et al. utilized a STZ-induced diabetic rabbit model to investigate the *in vivo* effects of curcumin, administered at a dosage of 300 mg/kg/day for a period of 3 months. Concurrently, *in vitro* experiments employed Rat H9C2 cardiomyocytes as experimental models, with curcumin being applied at varying concentrations (0–18 μmol/L). The experimental results indicated that curcumin significantly ameliorated the disarray in myocardial cells induced by diabetes, reduced myocardial cell edema, diminished lipid deposition between tissues, decreased the expression of collagen in myocardial tissue, and attenuated the severity of myocardial fibrosis. *In vitro*, the optimal concentration of curcumin was determined to be 10 μmol/L. Nuclear translocation of Nrf2, as detected by fluorescence co-localization, revealed that curcumin facilitated the nuclear import of Nrf2 and enhanced the expression of HO-1. This upregulation reduced the accumulation of intracellular reactive oxygen species in cardiomyocytes, mitigated GPX4 depletion, and hindered the progression of glucose-induced ferroptosis. Furthermore, curcumin also curtailed the excessive loss of GPX4, inhibited ferroptosis in cardiomyocytes, and ultimately improved the condition of DCM (Wei et al., 2022). These findings provide preliminary evidence suggesting that PCs can influence the course of diabetes and its complications by regulating ferroptosis-related pathways.

#### 4.2.3 Terpenoids

Terpenoids, the most diverse and abundant class of plant-derived chemicals, exhibit a broad spectrum of biological activities, including antiviral, anticancer, antioxidant, anti-inflammatory, and hypoglycemic properties (Wang et al., 2018). Ferroptosis, a form of regulated cell death, is characterized by increased lipid peroxidation and the accumulation of toxic lipid peroxides, which are believed to function as toxic ROS (Lata et al., 2011). Diabetic nephropathy, a complication of both type I and type II diabetes, is associated with elevated ROS levels. Hyperglycemia-induced mitochondrial damage leads to a significant accumulation of ROS, triggering oxidative stress, a key contributor to podocyte injury. Oxidative stress is thus recognized as a major driver in the development of diabetic nephropathy. The System Xc^−^/GPX4 pathway serves as a critical antioxidant defense system against oxidative stress (Chen et al., 2021b). GPX4, an enzyme, catalyzes the conversion of harmful intracellular lipid peroxides into non-toxic lipid alcohols using glutathione, effectively preventing ROS chain reactions and ferroptosis (Ursini et al., 2022). Conversely, the inhibition of System Xc^−^ activity by regulating SLC7A11 expression can lead to glutathione depletion and ferroptosis. Therefore, reducing and preventing ROS accumulation while enhancing GPX4 expression are crucial strategies for mitigating ferroptosis. Cheng *et al.* administered ginkgolide B (GB) at varying dosages (100, 200 mg/kg/day) via gavage to db/db mice for a period of 12 weeks. Metformin (MET), dosed at 200 mg/kg/day, served as a positive control. The experimental outcomes indicated that treatment with GB and MET resulted in a significant amelioration of renal pathologies. Complementing the *in vivo* findings, the study also encompassed *in vitro* investigations. MPC5 cells were exposed to GB at concentrations of 20, 40, and 80 μM for 24 h. It was observed that GB treatment led to a marked decrease in the production of ROS within the MPC5 cells, with the 80 μM concentration demonstrating the most potent antioxidant activity. To ascertain whether GB’s protective effects on MPC5 cells are mediated through the inhibition of ferroptosis, the study assessed the expression profiles of ferroptosis-associated markers, including TfR1, FTH1, and GPX4. The data revealed that GB treatment downregulated TfR1 expression while upregulating the levels of FTH1 and GPX4. These findings suggest that GB mitigates oxidative stress and ferroptosis by suppressing ROS accumulation and preventing GPX4 ubiquitination, thus conferring renal protection against ferroptosis and oxidative stress-induced damage, and potentially improving the prognosis of diabetic nephropathy (Chen J. et al., 2022). These findings collectively suggest that terpenoids hold promise as novel therapeutic candidates for diabetes and its complications by regulating ferroptosis-related pathways.

#### 4.2.4 Alkaloids

Alkaloids, a diverse group of naturally occurring secondary metabolites, are widely distributed across various organisms. Their structural complexity makes them a valuable source for discovering lead compounds with various pharmacological activities (Kundu, 2015). They are commonly present in different plant parts, such as roots, stems, leaves, and fruits, and exhibit various biological properties, including antibacterial and analgesic effects. Interestingly, research has revealed that certain alkaloids also possess antitumor properties. HO-1 is a stress-inducible protein that plays a vital role in the cellular defense system against oxidative damage. Its transcription is regulated by Nrf2. When Nrf2 protein levels increase, it promotes the gene transcription of HO-1 protein. Consequently, upregulating *Nrf2-HO-1* mRNA expression can potentially mitigate ferroptosis-related mechanisms (Amin et al., 2021; Hui et al., 2018). Berberine, a prominent alkaloid compound, has garnered significant research interest due to its pharmacological effects, including antioxidant, hypoglycemic, and anti-inflammatory properties. Studies have shown that blood sugar fluctuations associated with diabetes can lead to alterations in brain tissue microstructure and function, potentially contributing to the development of diabetic peripheral neuropathy. Huang *et al.* established an *in vitro* model using HT22 cells induced by Erastin. This study did not include *in vivo* experiments and lacked a positive control group. After pretreatment with different concentrations (30, 60 μM) of berberine for 2 h, the cells were treated with 0.5 μmol/L Erastin for 8 h. The results showed that high doses berberine (60 μM) can reduce the levels of active iron and ROS in HT22 cells, a cell line commonly used in neuropathy studies. Berberine can also modulate the expression of *Nrf2-HO-1/GPX4/ACSL4/PTGS2* mRNA, thereby reducing ferroptosis-induced apoptosis in HT22 cells triggered by Erastin, a ferroptosis inducer. This finding demonstrates the potential of berberine to improve diabetic peripheral neuropathy through the regulation of ferroptosis-related mechanisms (Huang Q. et al., 2022). These findings highlight the potential of alkaloids as therapeutic agents for diabetes and its complications by targeting ferroptosis pathways. This opens new avenues for exploring ferroptosis regulation as a future strategy for managing diabetic complications.

#### 4.2.5 Lignans

Lignans are naturally occurring dimeric compounds derived from cinnamic acid and its derivatives. They also contribute to the formation of lignin, a crucial polymer component of plant cell walls. Lignans exhibit a diverse range of biological activities, including antitumor, antiviral, hepatoprotective, immunosuppressive, antiplatelet, and cardiovascular benefits. Research has shown that certain lignans possess potent antioxidant and anti-inflammatory properties (Osmakov et al., 2022). Reducing oxidative stress is a critical therapeutic strategy for mitigating myocardial ischemia/reperfusion (MI/R) injury in diabetes (Zhang et al., 2018). Silent information regulator 2-related enzyme 1 (SIRT1) is a nicotinamide adenine dinucleotide (NAD^+^)-dependent histone deacetylase that plays a key role in regulating cellular responses to injury, including antioxidant defense and apoptosis (Yue et al., 2016). Nrf2 is a transcription factor that orchestrates the basal and stress-induced activation of numerous cytoprotective genes (Senyuk et al., 2021). Both SIRT1 and Nrf2 are recognized as critical regulators in diabetic patients experiencing MI/R injury. Activation of the SIRT1-Nrf2 signaling pathway has been shown to reduce oxidative stress and apoptosis (Zhang et al., 2018). Honokiol (HKL) is a lignan known for its potent antioxidant effects. It can inhibit oxidative stress by scavenging free radicals, preventing lipid peroxidation, and enhancing the activity of antioxidant enzymes. Consequently, HKL reduces peroxides in the body and exerts an anti-lipid peroxidation effect, thereby mitigating inflammatory responses (Li Y. et al., 2022). Subsequent investigations demonstrated that the oral administration of honokiol (5 mg/kg/day) for a period of 7 days can effectively activate the SIRT1-Nrf2 signaling pathway. This activation is associated with the mitigation of oxidative stress, attenuation of cellular apoptosis, and ultimately leads to the amelioration of myocardial injury in type I diabetic rats. However, the therapeutic benefits were partially abrogated by the Sirt1 inhibitor EX-527, suggesting a pivotal role for the SIRT1-Nrf2 axis in honokiol’s cardioprotective mechanisms (Zhang et al., 2018). These findings collectively suggest that lignans have the potential to influence the course of diabetes and its complications by regulating mechanisms associated with ferroptosis.

#### 4.2.6 Quinones

Quinones, a diverse class of organic compounds, are widely distributed in nature. They are found not only in a variety of plants but also in fungi, bacteria, and animals. Quinones exhibit a broad spectrum of potential biological activities, including antioxidant, anti-inflammatory, antibacterial, antimicrobial, and anticancer properties. They can be further categorized into four main types: benzoquinone, naphthoquinone, phenanthraquinone, and anthraquinone. Natural phenanthraquinones are classified into two subtypes: o-quinones and p-quinones. Notably, several phenanthraquinone derivatives isolated from the roots of *Salvia miltiorrhiza* Bunge (Lamiaceae), a traditional Chinese medicinal botanical drug, belong to both o-phenanthraquinone and p-phenanthraquinone groups. Tanshinone IIA (TIIA) is a well-characterized example and one of the main active components of red root Danshen. Ferroptosis, a form of regulated cell death, is critically dependent on the enzyme ACSL4. Downregulation of ACSL4 expression can impede ferroptosis (He S. et al., 2022), thereby mitigating high glucose (HG)-induced injury in HK-2 human renal proximal tubule epithelial cells (Fang et al., 2023). Embryonic lethal abnormal vision-like protein 1 (ELAVL1) is a well-studied RNA-binding protein that acts as a post-transcriptional regulator, influencing cell proliferation, ferroptosis, and metastasis. In diabetic nephropathy, ELAVL1 expression exhibits abnormalities and is considered an independent risk factor for kidney disease progression. Upregulation of ELAVL1 may promote HG-induced glomerular mesangial cell injury by stabilizing NADPH oxidase four during the course of diabetic nephropathy (Chen HY. et al., 2021; Shi et al., 2020). Drawing from these observations, the research team has delved into the potential of modulating the ELAVL1-ACSL4 axis as a strategy to mitigate HG-induced ferroptosis in cells. MPC5 cells were subjected to TIIA treatment at concentrations of 0, 5, 10, and 20 μmol/L for a duration of 24 h. The results indicated that a 10 μmol/L concentration of TIIA was capable of partially suppressing HG-induced injury and ferroptosis in MPC5 mouse podocyte cells by specifically targeting the ELAVL1-ACSL4 axis. Furthermore, db/db diabetic mice were utilized to assess the regulatory effects of TIIA *in vivo*. Concurrently, HE and MASSON staining revealed that TIIA, when administered intraperitoneally at a dosage of 10 mg/kg for 12 weeks, could significantly alleviate renal tissue damage. Additionally, the levels of pro-inflammatory cytokines IL-6, IL-1β, TNF-α, and the expression of ACSL4 were markedly reduced following TIIA treatment. Collectively, these findings suggest that TIIA may impede the progression of diabetic nephropathy by modulating ACSL4 activity *in vivo* (Zhu et al., 2024). Besides, a positive drug group was not set in this study, which is the limitation of this study. Studies have shown a strong link between diabetes mellitus and the development of atherosclerosis. This association involves several factors: dyslipidemia caused by diabetes, hyperglycemia-induced formation of AGEs, increased oxidative stress, and inflammation. Furthermore, endothelial cell damage due to lipid peroxidation-induced ferroptosis is recognized as a key contributor to atherosclerosis. Research has revealed a promising therapeutic strategy: TIIA can protect human coronary artery endothelial cells (HCAECs) from ferroptosis by regulating their intracellular redox state. Specifically, the studies suggest that TIIA exerts its protective effect by activating the Nrf2 pathway. Nrf2 is a crucial transcriptional regulator of genes that counteract ferroptosis. Activation of the Nrf2 signaling pathway is essential for maintaining a balanced redox environment within cells. By activating the Nrf2 pathway, TIIA effectively inhibits ferroptosis in HCAECs. This mechanism involves increasing the accumulation of Nrf2 in the nucleus and eliminating excessive ROS production. These findings suggest that TIIA has the potential to be a novel therapeutic strategy for treating atherosclerosis (Yang et al., 2023b). In conclusion, quinones can potentially mitigate diabetic complications and improve associated symptoms. This effect is likely mediated by their ability to regulate ferroptosis-related proteins and factors, including the ELAVL1-ACSL4 axis and Nrf2 expression. These findings suggest quinones as promising candidates for developing novel therapeutic strategies for diabetic complications.

## 5 Summary and outlook

**FIGURE 4:**
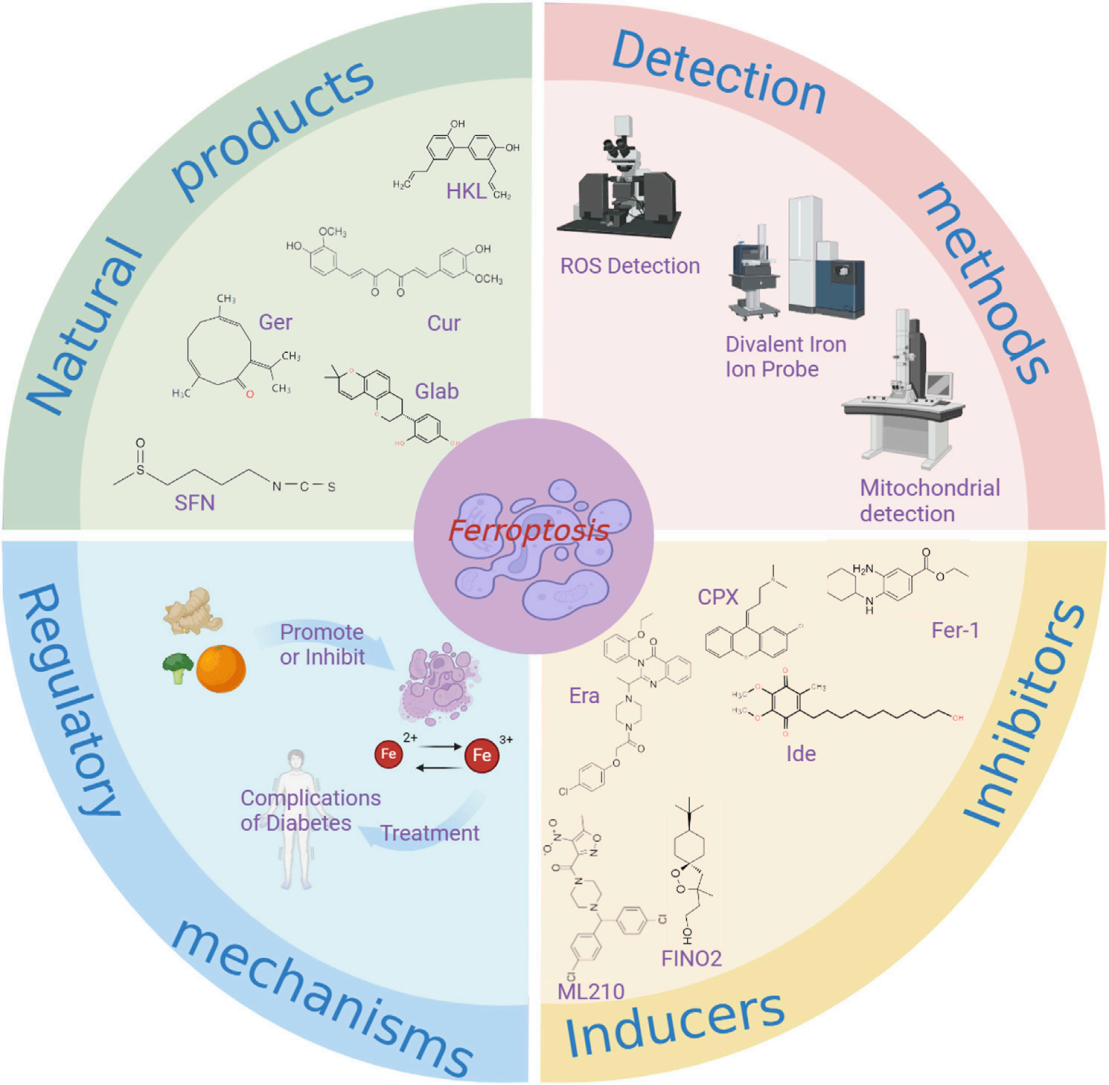
The relationship between ferroptosis and natural products in the treatment of diabetic complications. Cur, Curcumin; HLK, Honokio; Ger, Germacrone; Glab, Glabridin; SFN, Sulforaphane; Ide, idebenon; Era, erastin.

Ferroptosis, a form of regulated cell death triggered by iron-dependent lipid peroxidation, has recently been identified (Wu et al., 2020). Emerging research suggests that natural products can modulate ferroptosis and play a crucial role in managing diabetic complications (Han et al., 2022). This article examines novel approaches to treating diabetes and its associated complications by targeting ferroptosis with natural products (Figure 4). The discovery of ferroptosis has the potential to enhance treatment efficacy for diabetes using certain natural products, offering promising solutions. The article discusses methods for detecting ferroptosis, integrated information from natural product databases, investigates the interplay between natural products, ferroptosis, and diabetic complications, outlines the mechanisms of action of select natural products in managing diabetic complications, and charts a new research and treatment path for diabetic complications (Table 5).

**TABLE 5:** The case of natural products regulating ferroptosis on diabetic complications.

Natural product	Compound characterization	Form	Animal/cell models	Dose	Mechanism of action	Impact on complications	References
Glabridin (Glab)	Bioactive components of licorice botanical drug	flavonoids	Animal: STZ-induced SD rat	Animals: 50 mg/kg/day via ip injection for 4 weeks	Increasing SOD and GSH activities and reducing MDA and iron concentrations and TFR1 Expression by GPX4, SLCTA11, and SLC3A2	Diabetic nephropathy	Tan et al. (2022)
Nobiletin (Nob)	Important active ingredients in citrus fruits	flavonoids	Animal: STZ-induced SD rat Cells: H9C2 cells	Animals: 5 mg/kg via ip injection Cells: 100 μM for 12 h	Reduces the expression of ACSL4 and NCOA4	Diabetic cardiomyopathy	Huang et al. (2023)
Puerarin (PUR)	Active constituents in the dried roots of *Pueraria montana* var. *lobata* (Willd.) Maesen & S.M.Almeida ex Sanjappa & Predeep (Fabaceae)	flavonoids	Animals: STZ-induced SD rat Cell: HBZY1 rat mesangial cells	Animals: 100 mg/kg/day via ig administration for 8 weeks Cells: 1, 3, 10, 20 μM for 24 h (Minimum effective concentration: 10 μM)	Inhibits ROS production	Diabetic nephropathy	Hou et al. (2024)
Quercetin (QCT)	Active ingredients in many fruits, vegetables, flowers and leaves	flavonoids	Animals: db/db mice Cells:HK-2 cells	Animals: 25, 100 mg/kg via ig administration for 12 weeks (five times per week) Cells: 6.25, 12.5, 25, 50, 100 μM for 24 h (Minimum effective concentration: 25 μM)	Down-regulating the expression of TFR-1 and up-regulating the expression of GPX4, FTH-1, and SLCTA11, and increases the levels of Nrf2 and HO-1 to activate the Nrf2/HO-1 signaling pathway	Diabetic nephropathy	Feng et al. (2023)
Curcumin	A low relative molecular mass compound in *Curcuma longa* L. (Zingiberaceae)	polyphenol	Animals: STZ-induced New Zealand rabbit	Animals: 300 mg/kg/day via oral administration for 12 weeks	Increases nuclear translocation of Nrf2 and expression of oxidative scavengers such as Gpx4 and HO-1, reduces excessive loss of Gpx4	Diabetic cardiomyopathy	Wei et al. (2022)
GingkgolideB (GB)	Important active ingredients in Ginkgo biloba *Ginkgo biloba* L. (Ginkgoaceae)	terpene	Animals: db/db mice Cells: MPC5 cells	Animals: 100, 200 mg/kg/day via ig administration for 12 weeks Cells: 5, 10, 20, 40, 80, 100, 200 μM for 24 h (Minimum effective concentration: 20 μM)	Inhibits ubiquitination of GPX4 by promoting the expression of ferroptosis markers GPX4 and FTH1, while suppressing the expression of TfR1, as well as intracellular iron content and ROS levels	Diabetic nephropathy	Chen et al. (2022a)
Platycodin (PD)	A saponin from the dried root of *Platycodon grandiflorus* (Jacq.) A.DC. (Campanulaceae)with pharmacological characteristics	terpene	Cells: HK-2 cells	Cells: 1, 2.5 and 5 μM for 24 h (Minimum effective concentration: 5 μM)	Up-regulating GPX4 expression, downregulates ACSL4 and TFR1 expression, and upregulates FTH-1 and SLC7A11 expression	Diabetic nephropathy	Huang et al. (2022c)
1,8 Cineole (1,8-Cin)	Main components in *Eucalyptus globulus* Labill. (Myrtaceae) oil	terpene	Animals: STZ-induced C57BL/6 mice Cells: ARPE-19 cells	Animals: 50, 200 mg/kg/day via ig administration for 8 weeks Cells: 2.5, 25 nM for 72 h (Minimum effective concentration: 2.5 nM)	Down-regulating TXNIP expression and up-regulating PPAR-γ expression	Diabetic Retinopathy	Liu et al. (2023)
Loganin	It is one of the main medicinal components in *Cornus officinalis* Siebold & Zucc. (Cornaceae)	terpene	Cells: RSC96 cells	Cells: 0.1, 1, 10, 50 μM for 24, 48 and 72 h (Minimum effective concentration: 1 μM)	Inhibits ROS generation and nuclear translocation of NF-κB	Diabetic peripheral neuropathy	Cheng et al. (2020)
Sulforaphane (SFN)	Natural antioxidants in cruciferous plants	sulfurous compound	Animals: STZ-induced C57BL/6 mice	Animals: 0.5 mg/kg for 5 days each week via sc injection for 16 weeks	Upregulates the expression of Nrf2 and its downstream genes NQO1 and HO-1	Diabetic cardiomyopathy	Zhang et al. (2014)
Honokiol (HKL)	Main active ingredients in *Magnolia officinalis* Rehder & E.H.Wilson (Magnoliaceae) bark with beneficial properties	lignin	Animals: STZ-induced SD rat Cells: RSC96 cells	Animals: 25, 50, 100 mg/kg/day via ig administration for 4 weeks Cells: 2.5, 5 and 10 μmol/L for 48 h	Reduces oxidative stress and inhibition of the AMPK/SIRT1/PGC-1α axis and changes in downstream gene expression profiles	Diabetic peripheral neuropathy	Hu et al. (2023)
Tanshinone IIA (TIIA)	Main components of the root of *Salvia miltiorrhiza* Bunge	quinones	Animal models: db/db mice Cell model: MPC5 cells	Animals:10 mg/kg each week via ip injection for 12 weeks Cells: 5, 10, 20 μmol/L for 24 h (Minimum effective concentration: 10 μM)	Inhibits HG-induced MPC5 cell injury and ferroptosis by targeting the ELAVL1-ACSL4 axis	Diabetic nephropathy	Zhu et al. (2024)
Emodin	Main active component of *Rheum palmatum* L. (Polygonaceae) root and rhizome	quinones	Cell model: HK-2 cells Animal model: STZ-induced SD rat	Cells: 40 μmol/L for 48 h Dose: NA/(12 weeks)	Enhancing the expression of Nrf2, GPX4, SLC7A11 and FTH-1 proteins, restores the oxidative stress system	Diabetic nephropathy	Ji et al. (2023)

However, existing research still has many shortcomings. First, there is a lack of specific biomarkers for ferroptosis. Ferroptosis biomarkers currently used in preclinical studies are nonspecific, present in other types of cell death and certain pathological conditions, and are not signatures unique to diabetic complications. In this rapidly evolving field, the lack of ferroptosis-specific biomarkers has been a long-standing bottleneck limiting the development of ferroptosis-targeted clinical applications. Current research can only indirectly prove the occurrence of ferroptosis by detecting ROS levels, LOOH, GPX4 activity, etc. (Tang et al., 2022). Exploring suitable biomarkers will promote the development of further *in vivo* research and clinical monitoring. Secondly, almost all studies are preclinical, and the models are limited to cell and animal models. It should also be noted that most of these experimental animal studies investigated only a single dose of the drug or relatively small samples. There are few studies on the association between ferroptosis and human diabetic complications, making it difficult to determine whether the inhibitory effect of natural products on ferroptosis is only targeted at certain groups of people, or generally targeted at most patients with diabetic complications.

Most studies on the regulation of ferroptosis by natural products are limited in scope, with little exploration of the interactions between natural products in the treatment of diabetic complications. Current research encounters numerous challenges, such as the uncertainty regarding whether ferroptosis specifically inhibits other cell death pathways and the potential side effects of natural products in diabetes treatment through ferroptosis regulation, necessitating extensive clinical trials (Vadarevu et al., 2021). Questions remain regarding whether ferroptosis is an independent response to metabolic imbalance or if the imbalance directly triggers ferroptosis (Huang Y. et al., 2022). The active or passive nature of ferroptosis achievement is also debated, with emerging evidence pointing to crosstalk between ferroptosis and other forms of cell death (Ke et al., 2023; Li et al., 2019; Zhong et al., 2022). Further investigation into this interplay is crucial for understanding mechanisms and developing treatments. Additionally, deeper exploration of the relationship between natural products and diabetic complications is warranted. While some studies suggest natural products can regulate ferroptosis, research in this area is still in its early stages. It is essential to determine how different natural products influence iron regulation and cell death pathways when treating diabetic complications. Further exploration is needed to understand whether these pathways of ferroptosis regulation by natural products can be interconnected into a comprehensive regulatory network. Future research should take a multifaceted approach to identify natural products with therapeutic potential for diabetic complications and investigate their impact on ferroptosis pathways.

At the same time, further research is needed to investigate whether the role of ferroptosis varies among different complications of diabetes, if ferroptosis is specific to certain complications, and if ferroptosis directly contributes to the development of complications. For instance, in diabetes-related atherosclerosis, it is important to explore how iron-induced oxidative stress and the Nrf2 signaling pathway, a key player in oxidative stress response, may be regulated by ferroptosis and its potential role in the pathogenesis of atherosclerosis (Zhang Q. et al., 2021). Several studies have assessed the positive impacts of selenium, zinc, and chromium supplementation on diabetic patients and animals (Fakharzadeh et al., 2019). Research has indicated that selenium utilization by GPX4 is essential in preventing hydroperoxide-induced ferroptosis (Ingold et al., 2018). These findings collectively emphasize the importance of considering the regulation of ferroptosis in the treatment of diabetes-related complications.

Finally, despite a thorough literature search, our review has some limitations. Firstly, due to the scarcity of studies on natural products regulating ferroptosis in diabetes and its complications, the information on natural products in this article may be incomplete. It remains uncertain whether natural products have additional effects on the human body beyond treating diabetic complications. Secondly, all the studies analyzed in this review were basic experimental studies; no published clinical trials were included, and there was a lack of clinical data to support the findings. Nevertheless, we posit that exploring the role of natural products in regulating ferroptosis could offer novel insights for preventing and treating diabetic complications.
